# A brief primer on microRNAs and their roles in angiogenesis

**DOI:** 10.1186/2045-824X-5-2

**Published:** 2013-01-16

**Authors:** Sudarshan Anand

**Affiliations:** 1 Moores UCSD Cancer Center, 3855 Health Sciences Drive #0803, La Jolla, CA, 92093, USA

**Keywords:** Angiogenesis, microRNA networks, miR-126, miR-132, p120RasGAP, NOTCH, Ocular angiogenesis

## Abstract

Development of the vasculature is a complex, dynamic process orchestrated by a balance of pro and anti-angiogenic signaling pathways. The same signaling pathways are mis-regulated and exploited during pathological angiogenesis in cancer, inflammation and cardiovascular diseases and contribute to disease progression. In the last decade, small non-coding RNA molecules termed microRNAs (miRs) have emerged as key regulators of several cellular processes including angiogenesis. It is becoming clear that miRs function in complex networks and regulate gene expression both at the mRNA and protein levels thereby altering cellular signaling responses to specific stimuli. In the vasculature, miRs can function either in a pro-angiogenic manner and potentiate angiogenesis or act as anti-angiogenic miRs by enhancing cell death and decreasing endothelial proliferation. This review aims to provide an update on how microRNAs regulate gene expression and illustrate miR function in the vasculature with a discussion of potential applications of miRs as anti-angiogenic therapeutics.

## 

Vascular endothelium is a highly complex organ with a growing list of sophisticated functions. Endothelial cells have been implicated in diverse functions ranging from release of cytokines during influenza infections [1], maintenance of hematopoietic stem cells [2], to adipogenesis of both white and brown fat cells [3]. Suffice to say that endothelial cells have moved beyond the label of ‘cells lining the blood vessels’. Given such a broad array of functions, it is not surprising that there are multiple signaling networks [4] with several checks and balances that control endothelial responses to external perturbations. Over the last few years, microRNAs have emerged as critical regulators of signaling pathways in multiple cell types including endothelial and perivascular cells. There are several excellent reviews that have summarized the different miRs involved in vascular function during development and disease [5-7]. Here, I will focus on providing a broad framework to understand miR function and illustrate features of specific miRs in the regulation of angiogenesis.

## How are microRNAs made?

MicroRNAs (miRs) are small 20-24 nucleotide long RNAs that regulate gene expression by binding to mRNAs. miRs represent about 1-2% of the genes in several species including mammals [8]. Current version of miRbase reports 1600 precursors and 2042 mature miR sequences for humans and 855 precursors and 1281 mature miRs for mice [9]. It is estimated that miRs could well regulate 30-50% of protein coding genes. A large portion of miRs is encoded in introns of genes or in intergenic regions. They are transcribed as a primary miR (pri-miRNA) transcript by RNA polymerase II in conjunction with specific transcription factors that bind to a region upstream similar to a protein coding gene. These miR transcripts are then cleaved by a heterodimer of dsRNA binding proteins, Drosha and DiGeorge Syndrome Critical Region 8 (DGCR8), releasing a 55-70 nt small RNA hairpin termed pre-miR. This RNA hairpin is then exported out of the nucleus by Exportin-5 and Ran-GTP. In the cytoplasm, another RNAse III enzyme, Dicer cleaves the pre-miRs to generate 22nt duplex RNAs. These are now mature miRs that get incorporated into RNA induced silencing complex (termed RISC) consisting of two major classes of proteins Argonaute and GW182. While this is the canonical pathway of miR biogenesis, recent research has shown several alternative pathways depending on cell type, organism and biological contexts [10,11]. For example, the loss of Dicer leads to a more significant phenotype in the brain and embryonic stem cells compared to the loss of either Drosha or DGCR8. Similarly, loss of Dicer in the vasculature has a more profound effect than the loss of Drosha suggesting there may be Drosha independent mechanisms of miR biogenesis [12]. These observations highlight the degree of complexity in the different RNA processing pathways.

## How do miRs work?

When miRs were initially described, it was proposed that miRs bind to target mRNAs using a seed sequence consisting of 6-8 continuous bases and a few more complementary bases along the miR sequence. In plants, miRs have almost perfect complementarity with the target mRNA and these targets undergo direct RNAse mediated cleavage [13]. However, the mammalian miRs rarely had perfect complementarity with their targets. Therefore, it was hypothesized that mammalian miRs suppressed translation of target mRNAs leading to a decrease in target proteins [14]. Interestingly, recent work based on mRNA arrays and ribosome profiling has shown that miRs can also cause mRNA decay in mammalian cells and this is likely due to deadenylation of the mRNA [15]. The relative contributions of mRNA decay and translation inhibition mechanisms in suppressing the target protein levels remains an area of active investigation [16].

## What do miRs do that proteins cannot do?

It has been postulated that miRs maintain robustness of gene expression by insulating biological systems against noise [17]. For example, miR-1 and miR-7 in Drosophila insulate muscle differentiation and photoreceptor determination from external perturbations [18,19]. While there are other regulatory mechanisms that control gene expression programs such as transcriptional regulators, epigenetic modifications etc, miRs offer a unique advantage by regulating gene expression in the cytoplasm. Moreover, there are miRs that could be upregulated rapidly in cells up to several thousand copies per cell thereby achieving greater potency than otherwise possible. There have been simulations that demonstrate that miR mediated feed forward loops are more effective than transcriptional repressors in buffering gene expression against external perturbations [20]. Finally, the fact that miRs can regulate multiple targets, often several of them in a single pathway, makes them a more effective tool to modulate gene expression in response to specific stimuli.

## Are miRs essential in the vasculature?

The importance of miRs in vascular development was apparent based on loss-of-function studies of Dicer. Dicer hypomorphic mice had severe vascular deformation in both the embryo and the yolk sac and died at E12.5-E-14.5 [21]. Similarly, loss of Dicer led to pericardial edema and impaired circulation in zebrafish embryos [22]. Moreover, endothelial specific deletion of Dicer in mice decreased postnatal angiogenesis in response to multiple stimuli [23]. Similarly, inducible deletion of Dicer in vascular smooth muscle cells in mice led to impaired contractility, smooth muscle differentiation and vascular remodeling [24]. Taken together, these early studies established that the loss of Dicer and the concomitant loss of miR processing activity caused significant structural and functional defects in the vascular compartment.

## Which miRs regulate vascular function?

The first study to report a putative role for specific microRNAs in endothelial cells was performed by Poliseno et al. The authors used microarray based profiling and identified the most abundant miRs in HUVECs [25]. They reported that several miRs were predicted to target angiogenic growth factor receptors and characterized miR-221/222 as negative regulators of angiogenesis. One of the earliest miRs reported to affect endothelial function *in vivo* was miR-126. miR-126 is the most abundant microRNA in endothelial cells. Loss-of-function studies in mice and Zebrafish highlighted that miR-126 is important for developmental and pathological angiogenesis and implicated Sprouty Related EVH Domain containing protein 1(SPRED1), a negative regulator of Vascular Endothelial Growth Factor (VEGF) signaling, as a critical target of miR-126 [26,27]. Interestingly, experiments in Zebrafish demonstrate that blood flow can upregulate miR-126 via activation of Klf2, a mechanosensitive transcription factor [28]. Png and colleagues reported recently that miR-126 regulates endothelial cell recruitment to metastatic breast cancer cells, *in vitro* and *in vivo*[29]. They showed that miR-126 downregulated a complex network of targets IGFBP2, PITPNC1a and MERTK and suppressed metastatic endothelial recruitment, metastatic angiogenesis and metastatic colonization. These experiments suggest that the non-endothelial miR-126 can still impact angiogenesis albeit in an anti-angiogenic manner.

While the majority of miRs that have been reported function as pro-angiogenic miRs, miR-17 ~ 92 cluster, a well-characterized group of miRs plays a critical role as a negative regulator of angiogenesis [30,31]. Bonauer et al showed that miR-92a has an anti-angiogenic effect partly through targeting the integrin subunit α5. Loss of miR-92a improved functional recovery after myocardial infarction and limb ischemia by enhancing blood vessel growth [30,31].

The miRs highlighted here are examples to illustrate the diverse ability of miRs in manipulating endothelial function. We and others have reviewed the many different miRs that play a role in vascular function [32,33]. Some of the prominent miRs in endothelial cells and smooth muscle cells, their functions and target genes are listed in the table below (Table 1).

**Table 1:** Summary of different miRs and their roles in vascular cells

**microRNA**	**Role**	**Target(s)**	**References**
Let-7 family	Increases EC proliferation, migration	TIMP-1	[34]
miR-126	Enhances VEGF signaling in ECs	SPRED1, PIK3R2, VCAM-1	[26,27,35]
miR-132	Enhances growth factor signaling	p120RasGAP, PTEN	[36,37]
miR-210	Stimulates tube formation and migration	EphrinA3, NPTX1	[38,39]
miR-221/222	Inhibits cell proliferation, Anti-angiogenic	C-kit, p27	[25]
miR-296	Inhibits growth factor receptor recycling and degradation	Hepatocyte-growth factor regulated tyrosine kinase substrate	[40]
miR-424	Stablizes HIF-1, proangiogenic	Cullin-2	[41]
miR-92a	Anti-angiogenic. Inhibition enhanced neovascularization in ischemic tissues	ITGA5	[30]
miR-143/145	Critical for VSMC differentiation	KLF4/KLF5	[42]
miR-26a	Increases VSMC proliferation	Smad-1/4	[43]

## miR-132 in endothelial and perivascular cells

We identified miR-132 as an angiogenic miR that functions by regulating p120RasGAP and Ras signaling pathways using a human embryonic stem cell vasculogenesis model and primary endothelial cells [36]. We demonstrated that reciprocal expression of miR-132 and its target p120RasGAP correlated with pathological angiogenesis in human breast tumors and in human hemangiomas. Targeted delivery of an anti-miR-132 decreased angiogenesis and tumor progression in mouse tumor xenograft models. Consistent with our observations in vascular endothelial cells, Lagos *et al* reported that miR-132 is upregulated with similar kinetics and plays a key role in lymphatic endothelial cells during viral infections [44]. More recently, Mulik et al showed that miR-132 expression was upregulated by a VEGF-A and IL-17 dependent mechanism after ocular infection with Herpes Simplex Virus (HSV) [45]. The authors demonstrated that *in vivo* silencing of miR-132 by the provision of anti-miR-132 nanoparticles to HSV-infected mice led to reduced corneal neovascularization and diminished lesions. Taken together, these observations indicate that miR-132 may play an important role in pathological neovascularization downstream of multiple triggers including tumor-derived growth factors, viral infections and inflammation.

Subsequent to our work, other groups have reported the function of miR-132 in perivascular cells. Natarajan and colleagues showed time- and dose-dependent up-regulation of miR-132/212 by Ang II through the Ang II Type 1 receptor [37]. They identified phosphatase and tensin homolog (PTEN) as a novel target of miR-132 and demonstrated that miR-132 induces monocyte chemoattractant protein-1 in rat VSMC. The authors demonstrated the presence of a positive feedback loop where phosphorylation of cAMP Response Element Binding protein (CREB) led to miR-132 transcription; miR-132 overexpression resulted in enhanced CREB phosphorylation via p120RasGAP downregulation. In addition, the authors observed that aortas from Ang II-infused mice displayed similar up-regulation of miR-132/212 and monocyte chemoattractant protein-1 and concluded that miR132/212 can serve as a novel cellular node to fine-tune and amplify Ang II actions in VSMC.

Another recent study [46] showed that miR-132 was constitutively expressed and secreted by saphenous vein pericytes and facilitates cardiac regeneration following myocardial infarction. Both CREB and miR-132 were activated by treatment of these cells by VEGF-B or hypoxia/starvation. The authors report that miR-132 is secreted by these pericytes and can act as a paracrine activator of cardiac healing, highlighting a non-cell autonomous role for miR-132.

## What triggers miR expression?

The vasculature is exposed to several stimuli and stressors during developmental and pathological angiogenesis that trigger distinct miR expression profiles. For instance, miR-210 and miR-424 have been shown to be upregulated in response to hypoxia [38,41]. We have observed specific miRs upregulated in response to angiogenic growth factors VEGF and bFGF [36]. Other studies have also documented miRs regulated by Notch pathway signaling [47] and cytokines such as IL-3 [48]. These stimuli subsequently trigger miR expression via classic signal transduction pathways and transcription factors. For example, miR-132 is transcribed by CREB in multiple cell types including endothelial cells, vascular smooth muscle cells and neurons [36,37,49]. Harris et al have shown that miR-126 is transcribed by Ets family members Ets-1 and Ets-2 [50]. Interestingly, Nicoli et al demonstrated that blood flow induced mechanosensitive transcription factor Klf2a can also transcribe miR-126 during aortic arch development in Zebrafish [28]. While the factors that induce miR expression have been widely characterized, regulatory sequences such as promoters for specific miRs have not yet been well defined and remain an area of considerable interest.

## How do miR-target networks regulate angiogenic signals?

While initial reports of miR function focused on one or two specific targets of miRs, it is becoming clear that the biology of miR-target interaction is much more complicated than simple RNA-RNA binding that is the basis of target prediction algorithms. Recent evidence suggests that evolution of conserved miR/target networks can exert powerful influence on biological functions [51]. Given these considerations, one can envision future work in understanding physiological roles of miRs will include systems level analysis of multiple miRs and targets.

**Figure 1:**
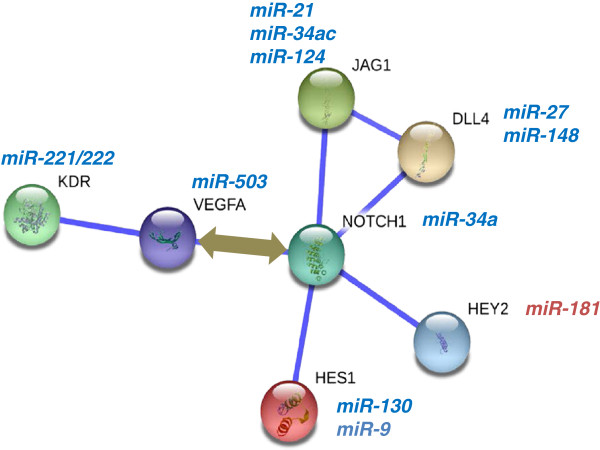
**A simple network of VEGF and Notch signaling regulated by microRNAs.** miRs identified in angiogenic screens [36] were cross-referenced with prediction programs for their ability to target specific nodes of a protein interaction network. Several miRs predicted to target angiogenic proteins were significantly downregulated in the screens (blue, italics). The illustration depicts how the loss of a few miRs that target positive regulators of angiogenesis can amplify a pro-angiogenic cascade.

For example, it is well established that VEGF and Notch signaling are key regulators of developmental angiogenesis. Mapping of a few receptors, ligands and transcription factors on this pathway using STRING database [52] shows how these molecules interact with each other. Our work identified several microRNAs that were regulated during growth factor signaling in endothelial cells and human ES cell vascular development. Some of these miRs from our screen have been predicted to target specific NOTCH or VEGF signaling components Figure 1. Interestingly, it appears that the miRs that were downregulated (blue, italicized) are predicted to target NOTCH or VEGF signaling components. This observation implies that downregulation of a few miRs can amplify a pro-angiogenic signaling network. Conversely, upregulation of miRs such as miR-132 and miR-126 can potentiate angiogenesis by targeting negative regulators of VEGF signaling such as p120RasGAP, SPRED1 etc. This network illustrates a cascade of miR transcription or decay events that can function in a positive feedback loop to amplify signals and sustain angiogenic responses Figure 1.

## Are miRs ‘druggable’ targets in human diseases?

Although miRs were discovered only about a decade ago, they have rapidly progressed to the verge of clinical utility. In fact, there are currently 124 clinical trials listed on microRNAs with 72 of them focused on cancer patients [53]. Many of these trials have focused on utilizing miR signatures for diagnostics across a spectrum of diseases. The first phase I trial of a microRNA therapeutic against HCV has shown excellent safety profile, good efficacy and has moved to Phase II trials highlighting that this novel class of drugs may have significant clinical potential [54]. Currently there are a number of different therapeutic strategies [55,56] that are being evaluated in preclinical models to inhibit miRs as described in Table 2.

**Table 2:** **Different strategies to inhibit miR function *in vivo***

**Reagent**	**Description**
AntagomiRs	Cholesterol conjugated 2’-O-Methyl antisense oligonucleotides
LNA-AntimiRs	Locked nucleic acid based antisense oligos
miR sponges	Oligos with multiple miR binding sites to act as competitive inhibitors
miR-masks	Oligos with gene specific sequences complementary to miR binding sites

Although these are exciting times for discovery of miRs and their applications, there are certain limitations to therapeutic targeting of miRs in the context of complex pathologies such as cancer.

1) Targeting anti-miR therapeutics to specific tissues. Naked injection of anti-miRs or cholesterol conjugated antagomiRs typically results in excellent delivery to the liver. However, targeting specific anti-miRs to tissues such as tumor vasculature or sites of ischemic injury is still challenging. Nanoparticles and other delivery vehicles with targeting peptides may be useful in delivering anti-miRs for specific applications [55].

2) The delivery of double stranded miR/miR mimics to tumor cells is still challenging and faces similar roadblocks as siRNA therapies. Recent work that shows that a single guide strand of RNA is sufficient to mediate RNA interference is exciting. This could lead to more efficient delivery of miRs to tumor cells *in vivo*[57,58].

3) The mechanism of action of miRs/anti-miRs is complicated by the number of potential targets that could be affected by a miR. There are discrepancies between prediction algorithms and experimental observations, not to mention cell type specific effects and differences in targets between human and mouse. Therefore, global analysis of mRNA and protein levels in cells/tissues that are targeted by the miR therapeutics may be warranted to understand the mechanism(s) of action.

## miR inhibitors as anti-angiogenic agents in ocular disease

miR inhibitors can be effective as simple oligonucleotides in ocular disease without the above mentioned requirements for specific delivery systems. Anti-angiogenic therapy, most notably, anti-VEGF antibody has shown significant therapeutic benefit in patients with Age-related Macular Degeneration (AMD) and consequently garnered several accolades including ‘*Science*-Breakthrough of the year 2006’ and the 2010 Lasker award [59]. There is proof-of-principle that targeting the VEGF pathway in the eye using single-strand RNA-based therapies can effectively block pathological neovascularization in humans, such as the VEGF RNA-aptamer Macugen/pegaptanib, which is approved for macular degeneration [60]. While the success of anti-VEGF approaches have been remarkable, it is not clear whether there are any long-term effects of targeting VEGF. Recently, Kurihara, Westenskow et al show that conditional deletion of *Vegfa* in adult mouse retinal pigmented epithelial (RPE) cells leads to vision loss and ablation of the choriocapillaris, the major blood supply for the outer retina and photoreceptor cells [61]. It appears that the loss of VEGF resulted in the downregulation of multiple angiogenic genes relevant for physiological and pathological angiogenesis. Therefore the authors conclude that endogenous VEGF provides trophic support necessary for retinal function.

Interestingly, our work has identified several promising miRs that are downstream of VEGF signaling pathways that can be pursued as alternatives to VEGF inhibitors in the eye. We demonstrated that anti-miR-132 is able to decrease developmental angiogenesis in neonatal retinas without affecting established vasculature [36]. Since the intraocular injection of anti-miR reagents selectively interferes with angiogenic endothelial cells that have upregulated miR-132 and not the normal quiescent vasculature, it is likely that this strategy will not have the global effects observed with VEGF ablation. In collaboration with Friedlander and colleagues, we have now observed that anti-miR-132 is a potent inhibitor of pathological neovascularization in the eye in various mouse models (Westenskow and Friedlander, submitted). It remains to be seen if long term use of anti-miR-132 and more broadly anti-angiogenic miRs, can circumvent some of the adverse effects associated with the lack of VEGF in the eye while effectively inhibiting pathological neovascularization.

## Conclusions

The emergence of miRs as regulators of gene expression has undoubtedly altered our understanding of how diverse stimuli affect function in the vasculature. miRs have been shown to alter specific signaling pathways that affect proliferation, differentiation, migration and cell survival in endothelial cells and smooth muscle cells. The challenges moving forward would be to integrate the different miRs and their target networks in the stimulus–response cycles of the vasculature and to understand how manipulating one or more miRs can alter cell function *in vitro* and *in vivo*. The therapeutic potential of these molecules as anti-angiogenic agents has been shown in multiple animal models and we may be just a few years away from using them to target aberrant angiogenesis in humans.

## Competing interests

The author declares that he has no competing interests.
